# Staged Norwood operation for patients with hypoplastic left heart syndrome and *cor triatriatum*: a report of two cases

**DOI:** 10.1186/s44215-024-00129-7

**Published:** 2024-03-07

**Authors:** Yukino Iijima, Takaya Hoashi, Takuro Kojima, Haruhiro Nagase, Yuji Fuchigami, Takaaki Suzuki

**Affiliations:** 1 Department of Pediatric Cardiac Surgery, Saitama Medical University International Medical Center, Hidaka, Saitama, 350-1298 Japan; 2 Department of Pediatric Cardiology, Saitama Medical University International Medical Center, Hidaka, Saitama, Japan

**Keywords:** Hypoplastic left heart syndrome, *Cor triatriatum*, Norwood operation, Hybrid stage I palliation

## Abstract

Two patients with hypoplastic left heart syndrome (HLHS) concomitant with *cor triatriatum* underwent the staged Norwood procedure following hybrid palliation. *Cor triatriatum* was diagnosed after birth in both cases. Case 1 with aortic stenosis and mitral atresia underwent bilateral pulmonary artery banding at 2 days of age. Subsequently, balloon atrial septostomy was conducted at 21 days of age due to hypoxia and increased intra-atrial pressure gradient. At 2 months of age, concomitant Norwood operation, *cor triatriatum* repair, and tricuspid valve repair were successfully performed. She has suffered from branch pulmonary artery hypoplasia since then; thus, Fontan operation was out of indication. Case 2 with aortic stenosis and mitral stenosis underwent bilateral pulmonary artery banding at 6 days of age. After that, the patient’s hemodynamic condition remained stable. The Norwood operation concomitant with *cor triatriatum* repair was performed at 2 months of age. However, during the procedure, massive pulmonary edema and hemorrhage occurred right after the initiation of cardiopulmonary bypass. She could not be weaned from cardiopulmonary bypass.

## Background

Hypoplastic left heart syndrome (HLHS) is characterized by the hypoplasia of the aortic arch and left heart structures, including the aortic valve, mitral valve, left ventricle, and left atrium. In cases where the left atrium is significantly underdeveloped, the pulmonary venous confluence and the true left atrium can appear as partially divided chambers, resembling *cor triatriatum*. Nevertheless, the occurrence of true *cor triatriatum* in conjunction with HLHS is infrequent and has been reported in only six patients [1–6].

Here, we present two surgical cases involving staged Norwood operations for patients with HLHS and *cor triatriatum*, following bilateral pulmonary artery banding with continuous prostaglandin administration. Both cases exhibited a transition type *cor triatriatum* from type 1 (intra-atrial communication from the posterior left atrial chamber to the right atrium, without an opening in the interatrial membrane) to type 2 (a restrictive opening in the interatrial membrane) [7].

## Case presentation

### Case 1

**Fig. 1:**
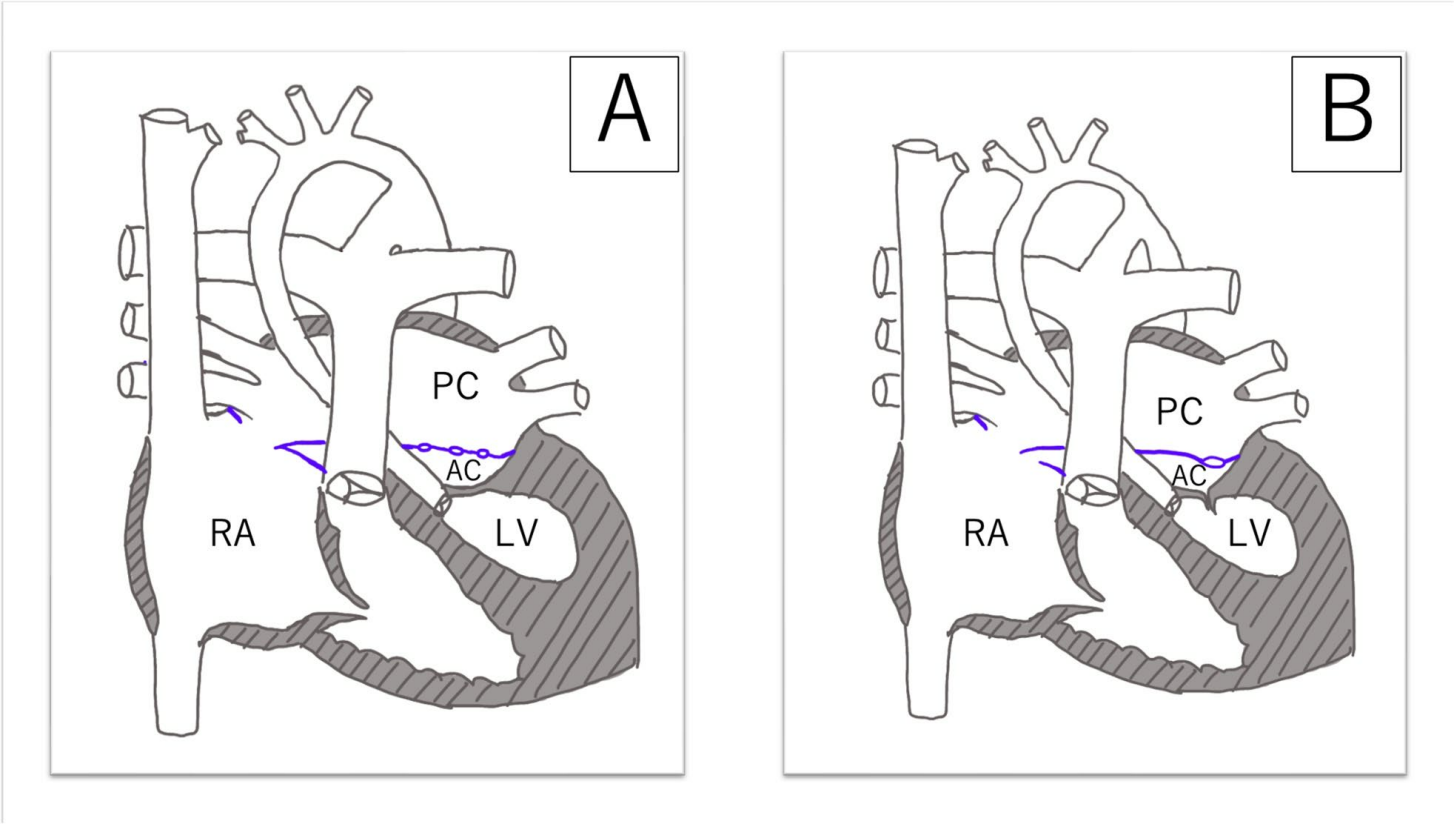
Schematic anatomy of patients 1 (**A**) and 2 (**B**). PC, posterior left atrial chamber; AC, anterior left atrial chamber; RA, right atrium; LV, left ventricle

**Fig. 2:**
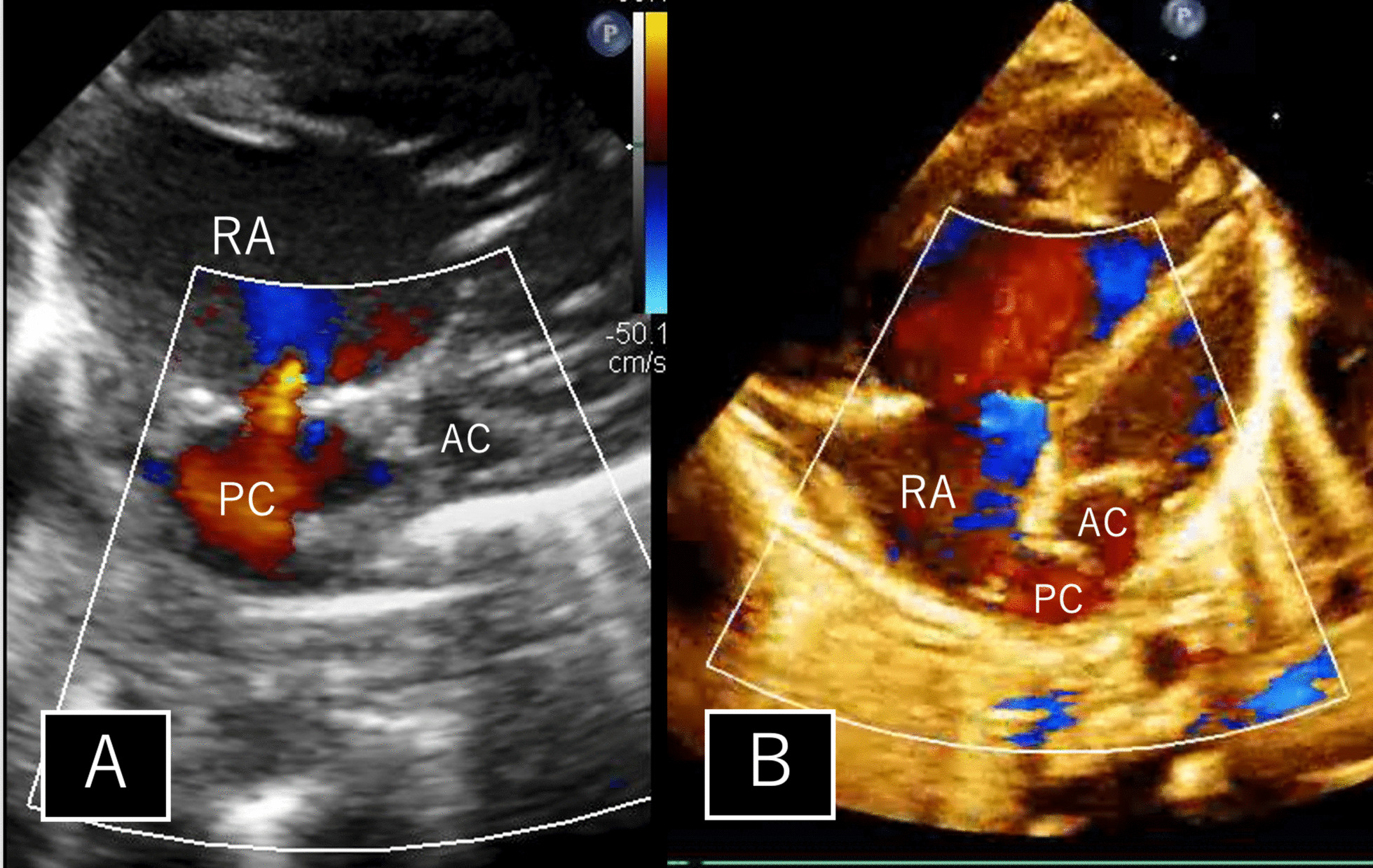
2D Doppler transthoracic echocardiogram images in patients 1 (**A**) and 2 (**B**). PC, posterior left atrial chamber; AC, anterior left atrial chamber; RA, right atrium

The first patient was delivered by cesarian section at 36 weeks of gestation, weighing 2069 g. Fetal echocardiography revealed aortic valve stenosis and mitral valve atresia, followed by the confirmation of *cor triatriatum* after birth (Fig. 1A). The left atrium was partitioned by a membrane, featuring a few minuscule apertures (< 2 mm each). The posterior left atrial chamber was connected to the right atrium through foramen ovale (Fig. 2A). The interatrial pressure gradient measured 2 mmHg. Bilateral pulmonary artery banding was conducted at 2 days of age. Subsequently, her oxygen saturation gradually decreased to 75%. Echocardiography detected a pressure gradient of 11 mmHg between the posterior left atrial chamber and the right atrium. At 21 days of age, balloon atrial septostomy was performed. Oxygen saturation improved to 85%, and the pressure gradient between the posterior left atrial chamber and the right atrium diminished to 1.4 mmHg. Thereafter, her condition remained stable, although moderate tricuspid valve regurgitation persisted.

At 2 months of age, weighing 2826 g, the patient underwent a concomitant Norwood operation, tricuspid valve repair, and repair of the cor triatriatum. Cor triatriatum repair was performed under cardioplegic arrest, after arch reconstruction, and before tricuspid valve repair. Through right atriotomy, the membrane between anterior and posterior left atrial chamber was resected via atrial septal defect. During the procedure, septum primum was also resected to enlarge the interatrial communication. Despite patch augmentation of both debanded branch pulmonary arteries, cardiopulmonary bypass could not be discontinued due to significant hypoxia. After upsizing the diameter of the right ventricle-to-pulmonary artery conduit from 5 to 6 mm, cardiopulmonary bypass could be weaned off.

At 5 months of age, the patient underwent stage 2 bidirectional Glenn procedure concomitant with redo tricuspid valve plasty and re-patch augmentation of the branch pulmonary artery. During this procedure, the right ventricle-to-pulmonary artery conduit was left with clipping. Subsequently, balloon pulmonary angioplasty was performed. At 24 months of age, Fontan eligibility was assessed by cardiac catheter examination; however, the superior vena caval pressure remained at 13 mmHg. The pulmonary vascular resistance measured 0.3 unit·m^2^. Fontan was not indicated at that time, and a re-patch augmentation of the tubular narrowing left pulmonary artery behind dilated aorta and removal of the right ventricle-to-pulmonary artery conduit was conducted at 25 months of age.

### Case 2

The second patient was delivered vaginally at 39 weeks of gestation, weighing 3020 g. Fetal echocardiography revealed aortic and mitral valve stenosis, and *cor triatriatum* was confirmed after birth (Fig. 1B). The abnormal membrane in the atrium appeared to have a slit-like opening. The communication between the posterior left atrial chamber and the right atrium was through small foramen ovale. Bilateral pulmonary artery banding was performed at 6 days of age. Subsequently, no significant pressure gradient was observed between the posterior and anterior left atrial chambers and the posterior left atrial chamber and the right atrium (Fig. 2B). Since then, the patient has remained in a stable condition with mild tricuspid valve regurgitation.

**Fig. 3:**
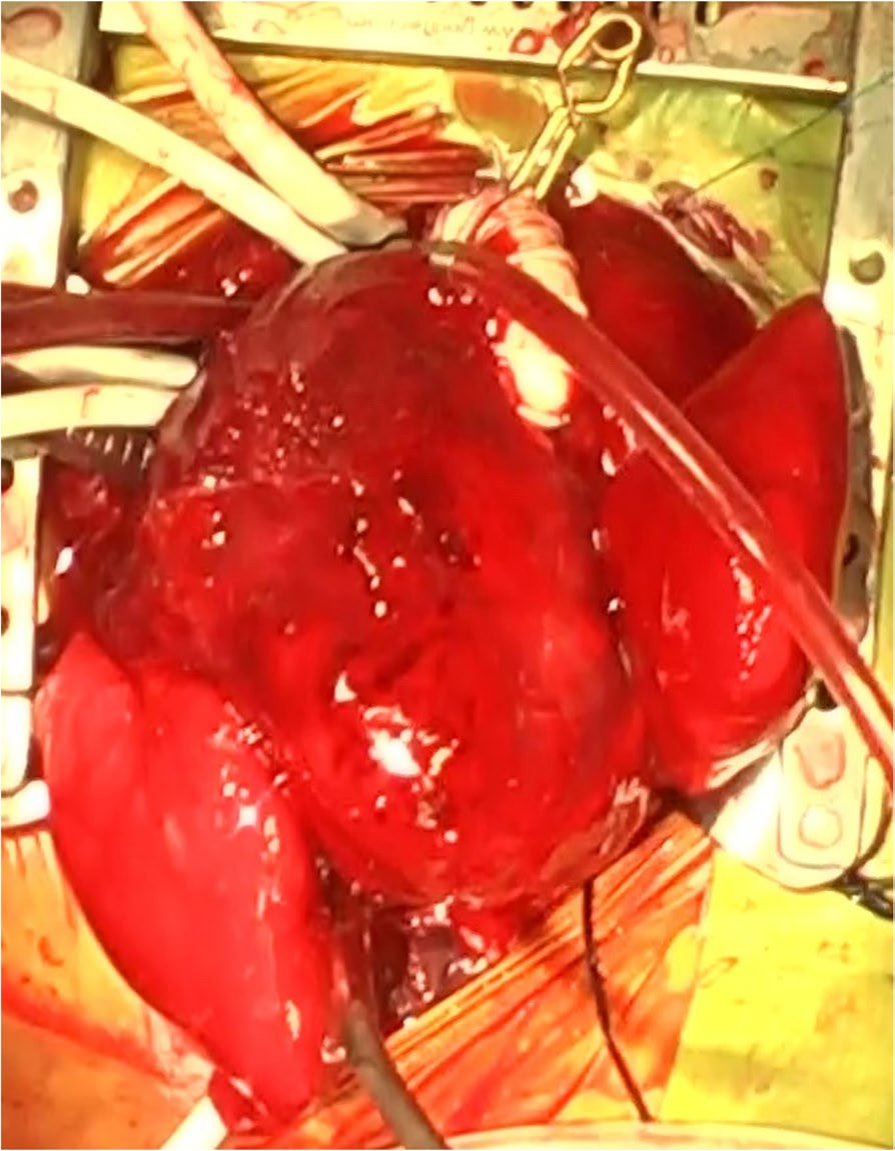
Intraoperative finding of Norwood and cortriatriatum repair in patient 2. Severely congested and expanded lung lobes and edematous heart were observed

At 2 months of age, weighing 3690 g, the patient underwent the Norwood operation and repair of *cor triatriatum*. During the Norwood procedure, the branch pulmonary arteries were debanded and temporarily clamped using a pair of vascular clips immediately after initiating cardiopulmonary bypass. However, while the blood was being cooled, severe pulmonary edema and hemorrhage occurred. At that time, central venous pressure was maintained to be under zero pressure. The heart was distended thereafter, which resulted in poor visibility in the operative field. Subsequently, venous drainage became insufficient, and whole-body edema was encountered. The central venous pressure exceeded 30 mmHg. Despite venous drainage cannulae were repositioned and replaced multiple times, venous drainage did not improve. Due to poor visibility in the operative field (Fig. 3), the process of arch reconstruction with an auto-pericardial patch, resection of the intra-atrial membrane, and insertion of the right ventricle-to-pulmonary artery conduit were extremely time-consuming.

She was transferred back to the intensive care unit with extracorporeal membrane oxygenation support. She died on post-operative day 3. Autopsy results confirmed the presence of lung edema and multiple spot-like hemorrhages.

## Discussion

Among the previously reported six patients with HLHS and *cor triatriatum* (Table 1), three patients underwent the Norwood operation. All three patients underwent the Norwood operation as the primary procedure, without preceding hybrid palliation. Among these cases, coexisting *cor triatriatum* was diagnosed during the Norwood operation in two patients. In one patient with an intact atrial septum, the muscular intra-atrial septum was resected, while in the other patient, the abnormal intra-atrial membrane was resected [1, 3]. The remaining patient was diagnosed prenatally and underwent the Norwood operation during the early neonatal period due to severely restricted opening of the intra-atrial membrane [2].

**Table 1:** Summary of reported HLHS and cortriatriatum cases

No.	Year	BAS	Surgery	Timing	Comorbidities	Cortri.	Prognosis
1	2007	–	+	Norwood		Muscle	Alive, BDG
2	2011	–	+	Norwood		Membrane	Alive, BDG
3	2001	–	+	Norwood		Membrane	Death
4	2009		Stillborn		Meckel-Gruber synd.		
5	2012		Best supportive care			Membrane	
6	2004	–	?			Muscle	Death

*BAS* balloon atrial septostomy: *Timing* the timing of surgical intervention for cortriatriatum: *Cortri* the type of cortriatriatum: *BDG* bidirectional Glenn

 During the Norwood operation in our second patient, a left atrial venting tube was inserted through the right upper pulmonary vein immediately after initiating cardiopulmonary bypass, because both inter- and intra-atrial communications between posterior left atrial chamber and right atrium or anterior left atrial chamber seemed restricted, despite no pressure gradient was noted by preoperative echocardiogram. However, because posterior left atrial chamber itself was so tiny, venting might have been ineffective due to sticking. Along with increase of both blood to the lung (containing hypoplastic pulmonary arterioles [8]) and returning from lung after bilateral pulmonary artery debanding, lung congestion and hemorrhage occurred, and the heart was subsequently distended. Finally, systemic venous drainage was mechanically obstructed, and whole-body edema became fatal.

In contrast to the second patient, the first patient underwent balloon atrial septostomy prior to the Norwood operation because a significant pressure gradient “fortunately” developed across the true right atrium and the posterior left atrial chamber, which contributed to her survival during the Norwood procedure. However, a relatively large-sized right ventricle-to-pulmonary artery conduit was required during the Norwood operation, likely due to mildly elevated pulmonary vascular resistance. Subsequently, she developed hypoplastic branch pulmonary artery, and therefore, the Fontan procedure has not been performed to date. Out of the three patients mentioned above who underwent the Norwood operation, one patient passed away before discharge, while the remaining two patients survived until stage 2 palliation. It remains unclear whether they proceeded to undergo the Fontan operation or not [1–3].

To prevent intraoperative pulmonary venous obstruction in patients with hypoplastic left heart syndrome and *cor triatriatum*, the feasibility of staged Norwood procedures seems limited [1–3]. If a staged Norwood approach is chosen, it is recommended to perform balloon atrial septostomy or surgical repair of *cor triatriatum* prior to the Norwood operation, regardless of the presence or absence of a significant intra-atrial pressure gradient under the condition of decreased pulmonary blood flow by bilateral pulmonary artery banding. Otherwise, primary Norwood strategy concomitant with repair of cor triatriatum should be a treatment of choice. According to the previous report, however, pulmonary lymphangiectasia, intimal thickening, and emphysema were frequently coexisted in HLHS patients with a restrictive foramen ovale, other than underdevelopment of small pulmonary arterioles [8]. To prevent such pulmonary parenchymal damages, fetal intervention “before Norwood operation during early neonatal period” should be also considered in patients with HLHS and cor triatriatum.

In summary, the staged Norwood operation was performed concomitantly with the repair of the transition type of *cor triatriatum* in two patients with HLHS, following bilateral pulmonary artery banding. Unfortunately, one patient did not survive. The surviving patient experienced underdeveloped branch pulmonary artery, and as a result, the Fontan procedure has not been performed thus far. Early management of pulmonary venous obstruction is crucial in the postnatal period *or fetal period* to mitigate the risk of intraoperative complications and pulmonary vascular resistance-related challenges in the future.

Of note, the authors declare that the entire manuscript was written in English by ourselves, but grammar and wording were corrected using large language models or ChatGPT.

## Data Availability

There are no additional data to disclose.

## Abbreviations

HLHS: Hypoplastic left heart syndrome
